# Teeth Whitening and Antibacterial Effects of *Juglans regia* Bark: A Preliminary Study

**DOI:** 10.1155/2021/6685437

**Published:** 2021-04-03

**Authors:** Riham Al-Rawi, Yusra Bashir, Aseel Mustafa, Mennatalla Omar, Noor AL-Rawi, Musab Saeed, Asmaa Uthman, Natheer H. Al-Rawi

**Affiliations:** ^1^Department of Oral & Craniofacial Health Sciences, College of Dental Medicine, University of Sharjah, Sharjah, UAE; ^2^Department of Pharmaceutical Sciences, College of Pharmacy, University of Sharjah, Sharjah, UAE; ^3^Department of Clinical Sciences, College of Dentistry, Ajman University, Ajman, UAE; ^4^Department of Diagnostic & Surgical Dental Sciences, College of Dentistry, Gulf Medical University, Ajman, UAE

## Abstract

**Objectives:**

Natural folk medicines with antimicrobial effects have been under investigation during the past decades. The aim of this study was to evaluate the teeth whitening and antimicrobial effects of ethanol extract of Persian walnut “*Juglans regia*” barks.

**Materials and Methods:**

Minimum inhibitory concentration (MIC) was determined using a broth microdilution assay which was conducted through a 2-fold serial dilution method, and a whitening experiment was done in vitro on extracted teeth, with a pH test being performed on 2-fold dilutions of the ethanol extract.

**Result:**

It was found that the MIC for *Enterobacter* and *E. coli* and *Staphylococcus* and *Pseudomonas* was found to be 5 mg/ml and 2.5 mg/ml, respectively. Both dilutions were found to be acidic, and the extract of *Juglans regia* bark also demonstrated the ability of teeth whitening.

**Conclusion:**

This study supports the use of *Juglans regia* bark as a natural product in dentistry because of the confirmed antimicrobial ability as well as its whitening effect. *Clinical Relevance*. Herb extract might be incorporated within commercially available kinds of toothpaste to enhance its whitening and antimicrobial effects.

## 1. Introduction

With the rising incidence of oral diseases and the demand for esthetically pleasing teeth, it is important to find successful and economical products that meet these needs. This calls for the significance of evaluating the effectiveness of oral health beliefs and traditional practices that uses medicinal plants. Oral health is a functional, structural, esthetic, physiological, and psychosocial state of well-being as defined by the ADA and is essential to the general health and quality of life of an individual [1].

In the human mouth, there are over 700 different bacterial strains that have been detected. These bacteria accumulate on hard and soft oral tissues and can be found in dental plaque niches [2]. A complex equilibrium between the host, the environment, and the microflora occurs in oral health. This is due to commensal bacteria that are suggested to be necessary to maintain oral health [3]. The symbiotic relationship between bacteria and the host is disrupted during illness, resulting in an imbalance in the microbiota of the oral resident, leading to the dominance of potentially pathogenic bacteria. Harmful metabolites, endotoxins such as lipopolysaccharides, exotoxins, and teichoic acid will be produced by bacteria that invade host cells. These bacteria change the environment, decreasing pH, thereby altering the dental plaque's microbial composition, and encouraging colonization of more anaerobic or acidogenic bacteria, resulting in unfavorable sequelae. As a consequence, there are dental caries and periodontal and many other oral diseases [4]. To minimize bacterial load in the mouth, it is, therefore, necessary to maintain oral hygiene, so brushing with fluoridated toothpaste twice daily and flossing should be practiced.

Using adjunctive products is also recommended like mouthwashes and special dentifrices according to their indications. One of the most common concerns with dental esthetics is the color of teeth. There has been a massive interest lately in getting whiter teeth. Teeth whitening products can be roughly classified into two main categories that include peroxide-containing bleaching agents and dentifrices. Bleaching agents can be used at home or done by a dentist. Typically, they are associated with tooth sensitivity and mild irritation of the soft tissue. On the other hand, dentifrices operate either by polishing, chemical chelation, or some other nonbleaching actions, so they are considered much milder but less effective. Both means of whitening require compliance and are relatively expensive, and since staining will inevitably occur, this requires retreatment and adds more to the cost.

Plants have been used for medicinal purposes for several decades, with an increased propensity for self-medication, and are still commonly used to this day. This is primarily due to the side effects of several synthetic medications, the emergence of resistance to many infectious disease medications, and the high cost of medication. These medicinal plants have active ingredients and can either be used directly without industrial processing or can be processed for purification and alteration, which can then be integrated into medicinal products. Some plants show promising potential, but many of them remain untested. *Juglans r*egia is one of these ancient traditional plants, which is identified with various names in many parts of the world, including Persian walnut, common walnut, English walnut, Carpathian walnut, and Madeira nut [5]. In various cultures, it also has various local words, such as derum, dandasa, and Sewak. Due to the advantages that it offers for oral health, the bark of this tree has traditionally been used as a teeth-cleaning twig. This involves cleaning of oral cavity, teeth whitening, which is commonly done in North Africa, South Asia, and the Middle East, as well as preventing the formation of calculus and bad breath. Not only did this plant serve dental purposes, but it was also used as a lip dye similar to lipsticks for cosmetic purposes [6].

Unfortunately, studies have not proved these advantages well. Therefore, the purpose of this study is to assess the efficacy of *Juglans regia* bark, especially in terms of in vitro whitening, antimicrobial effect, and pH level. Because of its lower cost, wide availability, and ease of use, it can be used as an adjunctive way to improve regular oral hygiene maintenance and provide esthetic enhancement.

## 2. Materials and Methods

This laboratory in vitro study was conducted at the Sharjah Institute of Medical Researches (SIMR), University of Sharjah, Sharjah, UAE.

### 2.1. Inclusion Criteria

1. Stem bark origin exclusively from Northern Iran
2. Sound anterior teeth without intrinsic stain

### 2.2. Exclusion Criteria

1. Stem barks from an unknown source
2. Teeth with extensive caries and/or with intrinsic stain

Stem bark collection and extraction were made as follows.

### 2.3. Collection and Management of *Juglans regia* Samples

The stem bark of Juglans regia was collected from a local market in Naif Souq, Dubai, United Arab Emirates, and was confirmed by the supplier that the origin is from Northern Iran. The plant was ground at a carpentry shop into fine powder. The powder was then weighed with a total weight of 820 grams.

### 2.4. Ethanol Extract Preparation

The powder was split into two equal masses and evenly deposited into two large flasks. Then, 99% ethanol was added till the powder was completely suspended. This was done to ensure the complete saturation of the ethanol by the powder. To allow the powder to dissolve, the mixture was shaken for 10 minutes and allowed to settle for 10 minutes. This resulted in the solution separating into 2 layers: a liquid ethanolic layer and a layer of undissolved powder precipitated into the bottom of the flask. Using a Buchner funnel, the liquid was filtered through double filter paper, and a vacuum suction was used to isolate the liquid from the undissolved powder. This method was carried out three times for each of the flasks. The resulting liquid was then evaporated using a rotary vapor machine to evaporate the ethanol, which resulted in a concentrated extract. The extract was then stored in vials and kept open for a few days in order to allow more ethanol to evaporate. To achieve a concentration of 0.25 mg/*μ*l, the extract was dissolved in DMSO (Dimethyl Sulfoxide), a highly polar organic liquid that is commonly used as a chemical solvent.

### 2.5. Identification of Extract Components

**Figure 1:**
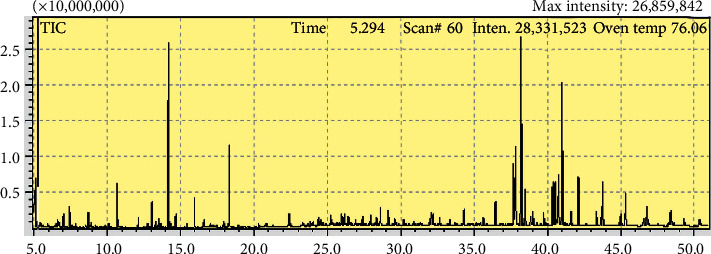
GC-MS analysis of *Juglans regia* extract.

As the samples dried up, 50 *μ*L of derivatization agent was added to each sample and left for about 30 minutes. 150 *μ*L of hexane solvent was later added and mixed with the existing solution. The solution was filtered by 0.22 um PTFE syringe filter and then transferred into GC vials, and they were ready to be processed by GC- MS (gas chromatography-mass spectrometry) to identify the extract components. The individual compounds in the extractives were identified by their retention time relative to known compounds, as shown in Figure 1.

### 2.6. Antimicrobial Test Preparation

The antimicrobial activity was evaluated against gram-positive *Staphylococcus*, gram-negative *Escherichia coli*, *Pseudomonas*, and *Enterobacteria*. The method used was 2-fold serial dilution with a duplicate done for each bacterium and their respective antibiotics on 96-well plates (12 × 8), starting with 5.0 mg/ml concentration. Two well plates each contain 2 bacterial samples, one containing *Staphylococcus* and *Pseudomonas* and the other containing *E. coli* and *Enterobacter*. DMSO was used as a negative control to ensure that bacterial growth inhibition was due to the active ingredient in the extract and not DMSO. Amikacin antibiotic was used as a positive control for *E. coli* and *Pseudomonas*, and Ciprofloxacin antibiotic was used for *Staphylococcus* and *Enterobacter*. Positive control was used to ensure that there was inhibition of growth, seen as lack of turbidity, and to compare these results to the effect of the extract on bacteria. Growth control consisting of bacteria suspended in broth was used to ensure that the broth was of nutritional value to the bacteria and resulted in growth. Media control consisting of broth only was used to ensure the purity of the broth.

Each of the incubated bacteria was suspended in saline to achieve an optical density of 0.5 McFarland units. 100 *μ*l of each suspended bacterium was added to test tubes containing 9.9 ml of broth to achieve a total volume of 10 ml of broth containing suspended bacteria.

A 96-well plate was used, each well plate has 12 columns and 8 rows (A-H) as shown in Tables 1 and 2, and since each plate was used for 2 bacteria, there was a mirror image in each well plate. The starting concentration was 5.0 mg/ml. To achieve this concentration, 2 *μ*l was taken from the extract (0.25 mg/*μ*l), but since it is a 2-fold serial dilution, 4 *μ*l was taken. In order to achieve a total volume of 100 *μ*l in A1 and B1, 96 *μ*l of broth containing suspended bacteria and 4 *μ*l of extract (0.25 mg/*μ*l) were added for every bacterium. 50 *μ*l of broth containing suspended bacteria was added to all the wells from A2 to A12 and B2 to B12. Using a micropipette, 50 *μ*l of A1 was transferred to the next well (A2), which already contained 50 *μ*l of broth containing suspended bacteria, resulting in a 1 : 2 serial dilution, which resulted in a 2.5 mg/ml concentration. This transfer step was repeated consecutively till A12, discarding 50 *μ*l from the last well to end up with the same volume in all the wells (50 *μ*l). Therefore, the sample concentrations were 5.000 mg/ml, 2.500 mg/ml, 1.250 mg/ml, 0.625 mg/ml, and so on. The duplicate that was done in row B was to eliminate experimental errors.

For C1and D1 wells, a positive control consists of 5 *μ*l of the complimentary antibiotic with 95 *μ*l broth containing suspended bacteria. 50 *μ*l of broth containing suspended bacteria was added to all the wells from C2 to C9 and D2 to D9. 50 *μ*l was taken from C1 and transferred to the next well (C2) which already contained 50 *μ*l of broth containing suspended bacteria. This transfer step was repeated consecutively till C9, discarding 50 *μ*l from the last well to end up with the same volume in all the wells. A duplicate was done in row D.

Other controls include negative control consisting of 2 *μ*l DMSO in 48 *μ*l of broth containing suspended bacteria in C10 and D10, growth control consisting of 50 *μ*l broth and 50 *μ*l of bacteria suspended in saline in C11 and D11, and media control consisting of 100 *μ*l broth in C12 and D12. The whole procedure was done in the other half of the same well plate for the other bacteria, as well as in the second well plate for the other two bacteria. The well plates were incubated for 24 hours at 37°C degrees and were then assessed visually for turbidity and carried into a microplate reader and scanned. The microplate reader measures optical density which gives values correlated with bacterial growth.

Due to *Juglans regia*'s intense color, turbidity could not be assessed for the first three concentrations that were tested against each bacterium; therefore, subculture analysis was done to confirm the presence or absence of bacterial growth at concentrations 5.000 mg/ml, 2.500 mg/ml, and 1.250 mg/ml. A 50 *μ*l from the selected wells was transferred into agar plates and allowed to incubate for a further 24 hours. The plates were then analyzed.

### 2.7. Whitening Experiment Preparation

Thirty sound human anterior teeth of varying shades with intact enamel layer and having no intrinsic staining were collected from the oral surgery clinic in the University Dental Hospital Sharjah and were stored in saline following extraction for this study. Fifteen teeth were randomly selected to be brushed with the extract, and fifteen were selected as control. Eight teeth were brushed with saline, and the remaining seven were only stored in saline with no brushing. All teeth were artificially stained by immersing them in a solution of tea prepared by dissolving 5 tea bags, 20 grams each, in 175 ml of water at 60°C. The tea was allowed to cool, and the teeth were then immersed for 12 hours. A baseline shade was captured using Vita EasyShade® Advance 4.0. A solution was made using the antimicrobial result of MIC (5 mg/ml) by dissolving 1 g of *Juglans regia* extract in 2 ml DMSO and 198 ml saline. The fifteen selected case teeth were brushed with a manual toothbrush for 2 minutes, using a concentration of 5 mg/ml of the prepared solution. The teeth were then stored in saline between each exposure to the extract. Eight of the control teeth were brushed for 2 minutes with saline and stored again in saline to confirm that whitening was due to the active ingredient in the *Juglans regia* plant and not due to the brushing. The other seven teeth were untouched and kept stored in saline, to ensure that there was no color change due to either storage in saline or delayed staining from the tea solution. This process was repeated twice daily for 7 days. The teeth were stored in saline between exposures to the *Juglans regia* solution for the duration of the experiment. A second reading was taken on the eighth day using Vita EasyShade® Advance 4.0 and noted. A comparison analysis in shade changes was conducted using paired *t*-test.

### 2.8. pH Test Preparation

A pH meter was used to measure the pH of different dilutions of the extract. A 2-fold serial dilution using saline was done on the extract of concentration 5 mg/ml: the MIC. 5 ml of saline was placed in each test tube, with exception of the first one, which contained 10 ml of the extract solution only. 5 ml from each test tube was transferred into the next resulting in 1 : 2 dilution. Each test tube was mixed well. Saline was used as a control to check the accuracy of the pH meter electrode. Following the instructions of the manufacturer, the electrode was rinsed with distilled water and dried using soft tissue to blot the liquid from the electrode before starting the experiment. The electrode was inserted in the first test tube (5 mg/ml), and the result was noted. The electrode was washed with distilled water between each reading to prevent contamination and to ensure the accuracy and precision of the readings. The same steps were followed for the other 3 test tubes, and their readings were noted.

## 3. Results

### 3.1. The Extractive Constituents of *Juglans regia*

Twenty fatty acids, three sterols, six organic acids, mineral acids, and one antibiotic were identified in the extract with the presence of nine alcohol-containing compounds and 3 phenolic compounds with eight sugars as seen in Tables 3 and 4.

### 3.2. Antimicrobial Test Results

After incubation of the two 96-well plates, the absence of turbidity was first visually assessed. The wells containing 5.0 mg/ml concentration of the extract and the following two dilutions were all dark in color. Lighter color and apparent turbidity were seen with the rest of the wells containing a decreasing concentration of the extract, the negative control, and the growth control, indicating bacterial growth. The absence of turbidity was seen with the highest three concentrations of the positive control and the media control, indicating the absence of bacteria.

A microplate reader was used to measure optical density at 570 nm. The lower the value given, the more the light transmission and less absorbance, indicating a low number of bacteria, and vice versa, as shown in Tables 1 and 2. All wells containing 5.0 mg/ml for all bacteria gave a value optical density value of 4 which is rounded to the nearest whole number. Wells containing 2.5 mg/ml extract gave an optical density value of 2 which is rounded to the nearest whole number for all bacteria. The remaining extract concentrations gave consecutively lower numbers as concentration decreased. Even though the highest concentrations were effective against bacteria, they showed high values due to the color of the extract which affected absorbance, compared to the positive control which showed lower values at high concentrations. Positive control with the highest antibiotic concentrations showed low optical density values which indicate that the antibiotics were effective against the tested bacteria. With further dilutions of the antibiotic, the subsequent optical density values given increased, as the antibiotic became less effective on bacteria. The negative control for all bacteria gave high values which indicate that there was no inhibition of growth due to DMSO. All media control gave optical density values below 0.4, which confirms that the broth used was not contaminated with bacteria.

A subculture analysis was done for wells containing the three highest concentrations 5.0 mg/ml, 2.5 mg/ml, and 1.25 mg/ml. The MIC for *Enterobacter* and *E. coli* was 5.0 mg/ml, whereas 2.5 mg/ml was found to be MIC for *Staphylococcus* and *Pseudomonas*.

### 3.3. Whitening Experiment

For the thirty tea-stained teeth, baseline and final readings of ADA bleaching guide, lightness, chroma, and hue were taken using Vita EasyShade® Advance 4.0. A two-tailed paired sample *t-*test was used to compare the values of the readings before and after conducting the experiment. Chroma and ADA bleaching guide of the teeth brushed using the extract showed significant difference as shown in Table 5.

### 3.4. pH Test

**Figure 2:**
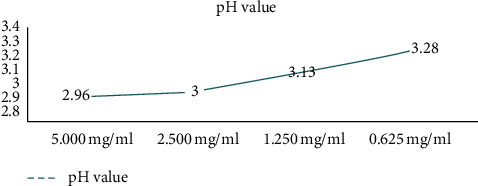
pH test results of different concentrations of the extract.

pH value was determined using a digital pH meter, was found to be acidic for all tested samples, and became less acidic as the extract becomes more diluted with saline. Detailed results are shown in Figure 2.

Saline, which was used as a control, had a pH of 5.5.

## 4. Discussion

Bacterial plaque is the most important cause of gingival inflammation and dental caries. This study shows that the bark of *Juglans regia* has an antimicrobial effect and may be used in oral hygiene practices to reduce bacterial load. *Juglans regia* ethanolic extract at high concentrations proved to inhibit the growth of *Staphylococcus*, *E. coli*, *Enterobacter*, and *Pseudomonas*, while it had little or no effect at low concentrations. A previous study reported that the aqueous extract of the *Juglans regia* bark showed a significant inhibitory effect against *Staphylococcus aureus*, *S. salivarius*, and *S. sanguinis,* but it had no effect on *S. mutans*. The ethanolic extract, however, had a significant antimicrobial effect on all four bacteria [7]. On the other hand, another study concluded that acetone extracts of *Juglans regia* are much more effective compared to aqueous extracts on salivary microbial flora samples from patients with dental caries [8]. The antimicrobial properties are attributed to the presence of cyclobutanol and 1,3-dioxolane-4-methanol, 2, 2-dimethyl in high percentage in the methanolic bark extract as shown in a study conducted by Ara et al. (2013) using gas chromatography-mass spectroscopy [9]. Furthermore, the antimicrobial properties may also be attributed to the presence of phenolic compounds, terpenoids, alkaloids, flavonoids, and steroids [9].

Juglan regia contains *β*-sitosterol, ascorbic acid, juglone, folic acid, regiolone, gallic acid, and quercetin-3-alpha-L-arabinoside [10]. The present findings have disclosed that the pH of all the 2-fold serial dilutions beginning with a concentration of 5 mg/ml is acidic and hence can be due to the presence of these compounds. Paradoxically, a study conducted by Alkhawajah (1997) aimed to test the effect of *Juglans regia* on the pH of the saliva in vivo by providing a piece of *Juglans regia* to volunteers to brush their teeth with it. Within 40 minutes, saliva pH showed an elevation where it reached a maximum of 7.55. This increase in the pH lasted for 3 hours. The change in pH was due to the stimulation of the salivary gland which amplified the salivary flow rate, washing out and diluting acids [11].

Teeth discoloration can be categorized into intrinsic and extrinsic staining. Intrinsic staining is related to genetics, intake of high levels of fluoride, and antibiotics during tooth formation, while extrinsic staining is due to environmental factors and colored compounds that are absorbed by the acquired dental pellicle on the surface of the tooth.

By showing a substantial difference in the chroma and ADA bleaching guide, the whitening experiment confirmed its efficacy on artificial extrinsic stain removal. This can be explained due to the acidic pH of the solution combined with the mechanical action of the brushing and using the abrasive powder. A study by Price et al. (2003) demonstrated the potential for acid-containing pastes to remove white, yellow, and brown stains from enamel by microabrasion action [12]. Upon intraoral use of the twig, the plant releases dark stains when wet by saliva which stains the soft tissues, including the gingiva. The perception of whiter teeth following the use of the *Juglans regia* twig may be experienced due to the contrast against the dark gingiva. Further studies are required to explore and identify the underlying mechanisms of whitening of *Juglans regia* extract as a solution and using the twig directly in vivo. Therefore, a long-term follow-up clinical trial with human subjects is required to show the difference in results between using the twig directly on the tooth surface, which is expected to cause abrasiveness, hence removing stains, and using the extract dissolved in a solution which was found to be acidic.

## 5. Conclusion

*Juglans regia* extracts are thought to have therapeutic potential which was confirmed by this study owing to the remarkable antimicrobial effect of *Juglans regia* bark against *Staphylococcus*, *Escherichia coli*, *Pseudomonas*, and *Enterobacter*, hence, demonstrating the possibility of incorporating it in a dental hygiene routine to prevent caries and periodontal diseases.

It was also concluded that this plant has an acidic pH. Therefore, additional studies are needed to be conducted to incorporate a buffer within the solution to neutralize the acidity of *Juglans regia*.

Finally, this study showed that *Juglans regia* has a potential capacity for teeth whitening, which may be similar in concept to enamel microabrasion. Further in vivo studies are needed to see if this plant can be used in the oral cavity without causing any harmful sequelae on teeth and their supporting structures, as well as to compare between methods of application and to know the possibility of incorporating extract solution into kinds of toothpaste.

### 5.1. Limitation of the Study

Due to the lack of extracted teeth required for the whitening experiment, the teeth were not selected based on the color they presented before their artificial staining which may have affected the results. This led to a small number of case teeth being selected and an even smaller control group. A larger number of teeth would have yielded more reliable results.

## Figures and Tables

**Table 1:** Microplate reader values for the 96-well plate containing *E. coli* and *Enterobacter* and their complimentary antibiotics (Amikan and Ciprofloxacin).

		5.0 mg/mL	2.5 mg/ml	1.25 mg/mL	0.625 mg/mL	0.313 mg/mL	0.156 mg/mL	0.078 mg/mL	0.039 mg/mL	0.020 mg/mL	0.010 mg/mL	0.005 mg/mL	0.002 mg/mL
		1	2	3	4	5	6	7	8	9	10	11	12
Staphylococcus	A	3.888	1.561	1.454	1.327	1.442	1.462	1.355	1.355	1.399	1.312	1.396	1.403
Staphylococcus	B	3.77	1.792	1.347	1.136	1.321	1.419	1.328	1.306	1.285	1.34	1.339	1.333
Ciprofloxacin	C	0.132	0.16	0.238	1.328	1.356	1.354	1.349	1.342	1.348	1.366	1.345	0.208
Ciprofloxacin	D	0.17	0.191	0.691	1.376	1.345	1.271	1.37	1.38	1.385	1.454	1.458	0.258
Amikacin	E	0.12	0.161	0.200	0.674	0.657	0.722	0.752	0.742	0.677	0.758	0.77	0.232
Amikacin	F	0.185	0.19	0.228	0.717	0.726	0.746	0.757	0.796	0.777	0.741	0.761	0.268
Pseudomonas	G	3.513	1.62	0.582	0.87	0.734	0.717	0.862	0.856	0.759	0.776	0.852	1.043
Pseudomonas	H	3.831	2.196	0.574	0.816	0.969	0.884	0.912	0.969	0.953	0.931	0.999	1.079

**Table 2:** Microplate reader values for the 96-well plate containing *Staphylococcus* and *Pseudomonas* and their complimentary antibiotics (Amikan and Ciprofloxacin).

		5.0 mg/mL	2.5 mg/ml	1.25 mg/mL	0.625 mg/mL	0.313 mg/mL	0.156 mg/mL	0.078 mg/mL	0.039 mg/mL	0.020 mg/mL	0.010 mg/mL	0.005 mg/mL	0.002 mg/mL
		1	2	3	4	5	6	7	8	9	10	11	12
*E. Coli*	A	4.077	2.394	1.616	1.31	1.21	1.083	1.064	1.049	1.073	0.953	1.026	1.119
*E. Coli*	B	3.909	2.367	1.604	1.298	1.044	0.988	1.038	0.98	0.984	0.837	1.074	1.093
Amikacin	C	0.278	0.579	0.915	0.961	0.999	0.914	1.016	0.946	0.971	1.018	1.053	0.362
Amikacin	D	0.325	0.601	0.858	0.947	1.015	0.923	0.966	0.996	0.944	0.972	1.033	0.408
Ciprofloxacin	E	0.694	0.845	1.205	1.222	1.31	1.301	1.253	1.241	1.307	1.549	1.322	0.225
Ciprofloxacin	F	0.191	0.966	1.226	1.191	1.23	1.154	1.153	1.11	1.051	1.456	1.212	0.261
Enterobacter	G	3.887	2.38	1.408	1.075	1.04	1.258	1.122	1.101	1.06	1.172	1.171	1.183
Enterobacter	H	3.868	2.231	1.384	1.084	1.066	1.236	1.056	1.096	1.086	1.073	1.091	1.06

**Table 3:** Fatty acids identified in *Juglans regia*.

Fatty acid	Retention time
4-methylvaleric acid	9.621
4-hydroxybutanoic acid	12.766
17-octadecynoic acid	14.298
Butanedioic acid	14.345
Nonanoic acid	15.299
Myristic acid	25.368
10,13-octadecadienoic acid, methyl ester	30.06
Linoleic acid ethyl ester	31.251
Pentacontanoic acid, ethyl ester	31.859
9,12-octadecadienoic acid	32.103
13-octadecenoic acid	32.33
11-methyltricosane	32.611
Stearic acid, TMS derivative	32.678
2-hydroxyethyl palmitate	34.142

**Table 4:** Sterols identified in *Juglans regia*.

Sterol	Retention time
Campesterol	47.187
Stigmasterol	47.52

Organic acid	
Lactic acid	8.898
Ethanimidic acid	**9.865**
Glycolic acid	10.68
Citric acid	24.722

**Table 5:** Teeth brushed with extract solution versus control groups.

Tested groups	Lightness				Hue				Chroma				ADA bleaching guide			
	Before	After	*T*-test	*P* value	Before	After	*T*-test	*P* value	Before	After	*T*-test	*P* value	Before	After	*T*-test	*P* value
	Mean ± SD	Mean ± SD			Mean ± SD	Mean ± SD			Mean ± SD	Mean ± SD			Mean ± SD	Mean ± SD		
Teeth brushed with extract solution (*N* = 15)	1.25 ± 0.76	0.35 ± 2.11	1.63	>0.05	0.69 ± 0.90	1.37 ± 3.23	−0.87	>0.05	−1.20 ± 1.09	3.02 ± 2.94	2.36	<0.04^*∗*^	23.33 ± 3.64	14.88 ± 7.71	4.26	<0.04^*∗*^

Teeth brushed with saline solution (*N* = 8)	0.70 ± 0.94	1.56 ± 0.97	−1.79	>0.05	1.26 ± 1.27	3.78 ± 2.79	−1.98	>0.05	−2.00 ± 1.65	−3.82 ± 0.84	1.76	>0.05	20.80 ± 4.37	17.80 ± 6.42	0.98	>0.05

Teeth stored in saline only (*N* = 7)	1.73 ± 1.06	1.63 ± 0.93	1.00	>0.05	2.07 ± 1.27	0.47 ± 0.32	1.96	>0.05	1.53 ± 0.23	−1.37 ± 0.40	−1.39	>0.05	27.33 ± 2.89	25.00 ± 1.37	3.50	>0.05

^*∗*^Significant difference at *P* < 0.05

## Data Availability

The data are available on request through the authors themselves.
